# Prospective randomised double‐blind trial: holmium laser lithotripsy with vs without Moses™ 2.0

**DOI:** 10.1111/bju.70446

**Published:** 2026-09-13

**Authors:** Kimberly A. Maciolek, Naren Nimmagadda, Aaron X. Lee, Amy M. Reed, Ryan S. Hsi, Tatsuki Koyama, Connor M. Forbes, Matthew S. Lee, Cyrus Chehroudi, Ezra J. Margolin, Ben H. Chew, Seth K. Bechis, Mitchell R. Humphreys, Michael E. Lipkin, Manoj Monga, Ojas Shah, Karen L. Stern, Roger L. Sur, Sri Sivalingam, Nicole L. Miller

**Affiliations:** ^1^ Vanderbilt University Medical Center Nashville TN USA; ^2^ University of Virginia Charlottesville VA USA; ^3^ Johns Hopkins University School of Medicine Baltimore MD USA; ^4^ Brook Army Medical Center San Antonio TX USA; ^5^ University of California Irvine Irvine CA USA; ^6^ University of California San Diego Health San Diego CA USA; ^7^ The Ohio State University Wexner Medical Center Columbus OH USA; ^8^ Glickman Urological and Kidney Institute Cleveland Clinic Cleveland OH USA; ^9^ Advent Health Urology Altamonte Springs FL USA; ^10^ Columbia University Irving Medical Center New York NY USA; ^11^ Mayo Clinic Phoenix AZ USA; ^12^ Duke University Health System Durham NC USA; ^13^ University of British Columbia Vancouver British Columbia Canada

**Keywords:** kidney calculi, ureteroscopy, laser lithotripsy, solid‐state lasers, workload, adult

## Abstract

**Objective:**

To assess the effect of Moses™ 2.0 (Lumenis Be Ltd., Yokneam Illit, Israel; Boston Scientific, Marlborough, MA, USA) for lithotripsy of renal stones of 8–20 mm in diameter on operative time, surgeon workload, and retropulsion.

**Subjects/Patients and Methods:**

In our multicentre, randomised, double‐blind clinical trial, patients were randomised 1:1 to standard or Moses 2.0 laser lithotripsy and stratified by stone size. Patients and surgeons were blinded to laser modality. Primary outcomes were total operative time as well as surgeon workload assessed by the National Aeronautics and Space Administration‐Task Load Index (NASA‐TLX). Secondary outcomes were stone retropulsion evaluated by blinded video review and postoperative clinical outcomes. Linear regression modelling was used for analysis.

**Results:**

A total of 153 patients (79 Standard, 74 Moses 2.0) with similar clinical, stone, and operative characteristics were evaluated. There was no significant difference in operative times between the Standard and Moses 2.0 groups (*P* = 0.73). Moses 2.0 was associated with lower effort, frustration, mental, physical, and temporal workload, as well as higher rated overall performance compared to the standard laser. Video review demonstrated no difference in rates of retropulsion (*P* = 0.13). Complication rates (Standard: 13%; Moses 2.0: 10%) and stone‐free rates (Standard: 51%; Moses: 2.0 55%) were comparable.

**Conclusion:**

Our study evaluated the impact of Moses 2.0 technology on high‐powered renal stone dusting, constituting the largest double‐blind trial of its kind to date. There was insufficient evidence to suggest any difference in operative times or clinical outcomes when using Moses 2.0 compared to standard laser lithotripsy. Moses 2.0 was associated with reduced surgeon workload, which has implicit impact on surgeon wellness.

**Abbreviations**
NASA: National Aeronautics and Space Administration
NASA‐TLX: NASA‐Task Load Index
REDCap: Research Electronic Data Capture
SFR: stone‐free rate
URS: ureteroscopy
YAG: yttrium‐aluminium‐garnet

## Introduction

Ureteroscopy (URS) with laser lithotripsy remains first‐line therapy for renal stones <20 mm in size [1, 2]. Its utilisation now approaches two‐thirds of all stone surgeries performed [3]. This trend is intuitive given URS is minimally invasive, efficient, and definitive with laser fragmentation of the stone under endoscopic visualisation. Despite its advantages, the mean URS treatment time is typically >1 h and leaves residual fragments >2 mm in 22% of patients [4]. The optimal method of laser stone fragmentation is debated, but a ‘dusting’ technique (i.e., applying low laser pulse energy at high frequency to ablate the stone and obviate the need for manual basket extraction of fragments) has gained popularity and been shown to have comparable stone‐free rates (SFRs) with shorter operative time compared to basketing [5].

However, dusting has been associated with increased operative effort. Dusting is performed with continuous, surface‐sweeping motion instead of focused direct laser energy is avoided to prevent stone fragmentation. This technique requires the stone to be stationary for maximal ablation efficiency, which is difficult to maintain with higher laser frequencies and respiratory movements. Long pulse‐width modulation was the first innovation counteracting the trade‐off between frequency and stone contact in which laser energy is more equally distributed over a longer emission interval; this minimises the peak power of the laser pulse, leading to less cavitation and therefore less mechanical fragmentation of the stone [6].

The next evolution in pulse modulation technology utilised the ‘Moses effect’, creating vapour bubbles through which laser energy can travel more efficiently to increase stone ablation and reduce retropulsion [7]. Lumenis (Lumenis Be Ltd., Yokneam Illit, Israel; Boston Scientific, Marlborough, MA, USA) was the first to optimise this laser phenomenon with ‘Moses™ Technology’ in their Lumenis Pulse™ P120H holmium:yttrium‐aluminium‐garnet (YAG) laser system, initially launching in 2017 and later upgrading to a second generation referred to as Moses 2.0.

There are few randomised controlled trials analysing the clinical benefits of the Moses effect in laser lithotripsy, especially Moses 2.0. Therefore, our aim was to perform a multicentre, randomised, double‐blind clinical trial to assess the clinical benefits of the Moses 2.0 effect. We hypothesised that Moses 2.0 technology would demonstrate benefits such as reduced operative time and surgeon workload compared to standard laser lithotripsy.

## Subjects/Patients and Methods

### Study Design

We performed a multicentre, randomised, double‐blind, clinical trial to further assess the effect of Moses 2.0 technology for holmium:YAG lithotripsy of renal stone burden totalling 8–20 mm in diameter. Members of the Endourology Disease Group for Excellence (EDGE) research consortium devised the randomised trial across six member sites in the United States (Vanderbilt University, Cleveland Clinic, University of California San Diego Health, Columbia University and Duke University, Mayo Clinic Arizona). Institutional Review Board approval was obtained at each site. The trial was registered on clinicaltrials.gov (NCT04505956). Enrolment occurred between January 2021 and May 2023. All patients provided written informed consent before participation. The study followed the Consolidated Statement of Reporting Trials (CONSORT) statement.

Adult patients aged ≥18 years and able to provide their own consent were eligible if undergoing URS for a solitary renal stone or additive maximal diameter of stones totalling 8–20 mm in size on CT. Stones were required to be at or above the level of the PUJ. Exclusion criteria included concomitant ureteric stones, ipsilateral ureteric stricture, prior ipsilateral upper urinary tract reconstructive procedures, prior radiotherapy to the abdomen or pelvis, neurogenic bladder or spinal cord injury, pregnancy, and untreated UTI. In situations of simultaneous contralateral URS, only one side was eligible for study inclusion; in these cases, the study side was required to be completed prior to transitioning to the non‐study side. The treating urologist determined the laterality for study in these cases.

### Surgery Technique

The URS was performed via standard technique by attending endourologists only. Trainees could only serve as assistant surgeons. Lumenis Pulse 120H Moses 2.0 holmium:YAG laser systems with 200‐μm D/F/L fibres were used for all cases. Fragments visually smaller than the laser fibre diameter indicated adequate dusting. Ureteric dilatation, use of a safety wire, and/or placement of a ureteric access sheath were performed at the discretion of the surgeon. In all cases, a ureteric stent was placed, and alpha‐blockers were prescribed postoperatively.

### Laser Settings

Both patients and surgeons remained blinded to the laser settings while a laser operator (not surgeon or assistant) retrieved the randomisation and set the laser machine accordingly. Initial settings were standardised for each group. Standard laser settings consisted of 0.2 J and 80 Hz (left pedal) and 0.5 J and 80 Hz (right pedal) with short pulse selected for both pedals. Moses 2.0 settings consisted of 0.2 J and 80 Hz on distance mode (left pedal) and 0.3 J and 120 Hz defaulted to contact mode (right pedal). Settings were adjusted at surgeon discretion, while blinding to treatment assignment was maintained, by increments of 0.1 J and 10 Hz without specifying a specific joule or hertz. Postoperatively, laser log files exported from the Lumenis Pulse P120 H Moses 2.0 laser generator were analysed for lasing time, number of times the surgeon pressed the laser pedal per procedure, percentage of time the laser was on vs off, and the total energy required to fragment stones during each procedure.

### Data Collected

Operative time was recorded as time from cystoscope in to out. Surgeons completed the National Aeronautics and Space Administration‐Task Load Index (NASA‐TLX) to measure surgeon workload at the end of each procedure. The NASA‐TLX consists of six questions addressing mental demand, physical demand, temporal demand, frustration, effort, and performance. Each item was marked on a 20‐point scale (low = 1, high = 20, Fig. S1) [8, 9, 10]. Surgeons were also surveyed, while blinded, to predict whether Moses 2.0 settings were utilised.

Video clips comprising the first and last 10–20 s of dusting were recorded to grade for retropulsion and ensure adequacy of dusting. Blinded video review was performed by two non‐study‐site‐affiliated endourologists (C.M.F. and M.S.L.) with retropulsion graded on a Likert scale (no retropulsion, some retropulsion, significant retropulsion). Residual stone fragments were visually estimated as no residual fragments or as fragments ≤1 mm, 1–2 mm, and ≥2 mm. Intra‐ and postoperative adverse events were evaluated. SFRs were determined by non‐contrast CT obtained at 6 weeks postoperatively. SFRs were defined as: Grade A = no residual stones; Grade B = ≤2 mm fragments; Grade C = 2.1–4 mm fragments; Grade D = >4 mm fragments. Data were stored in Research Electronic Data Capture (REDCap) application [11, 12].

### Sample Size Calculation

Based on a previous trial where dusting techniques were used [5], the mean (SD) operative time for dusting using standard laser settings was estimated at 35.9 (17.8) min. The study was originally powered to detect a 6‐min reduction in operative time with the Moses 2.0 laser group. A sample size of 146 patients per arm (total *n* = 292) was determined to provide 80% power. Allowing for a 5% attrition rate, the total enrolment target was set at 300 patients across participating sites.

During the course of the study, the rapid adoption of suction‐assisted URS fundamentally altered the clinical and technological landscape of endoscopic stone management. This evolution impacted both the relevance of procedural benchmarks, and feasibility of continued enrolment under the original assumptions. Accordingly, the study steering committee initiated an early evaluation of accumulated data. Recruitment was concluded with the final patient enrolled in March 2023. Interim analysis demonstrated nearly equivalent total operative times between the Moses 2.0 and Standard groups. Based on these findings, a re‐calculated sample size to detect a 3‐min reduction in operative time between the two groups with a power of 80% was >1200 patients‐ rendering continuation of the study impractical due to resource, time, and ethical constraints. Furthermore, ongoing recruitment under the existing protocol would have delayed patient access to emerging suction‐based technologies to treat their stones.

### Randomisation

Patients were randomised 1:1 to standard holmium laser lithotripsy or holmium laser lithotripsy using Moses 2.0 technology. Randomisation was performed prior to the procedure and stratified within each study site by renal stone size (8.0–12.0, 12.1–16.0, 16.1–20.0 mm). Block sizes of two, four, and six were used within each stratum to enhance blinding. The randomisation system was developed using R (R Foundation for Statistical Computing, Vienna, Austria) and implemented using REDCap. Concealment was performed with respect to the randomisation list.

### Statistical Analysis

Intention‐to‐treat analysis included all randomised patients. Demographic and perioperative variables were summarised by treatment group using median and quartiles for continuous variables and frequency and percentage for categorical variables. Clinical, stone, and surgical characteristics were compared using the chi‐square test or Wilcoxon rank‐sum test, as appropriate. For each primary outcome, a linear regression model was fitted, with treatment as the main predictor, and patient age, body mass index, Hounsfield units of the stone as measured by CT, number of stones, axial stone size category, use of stone repositioning with a basket, use of a digital ureteroscope, use of an access sheath, and dusting location (lower pole vs other) as adjusting covariates. An interaction term between treatment and stone size was included to assess whether the treatment effect differed by stone size. Secondary outcomes were analysed using the same modelling approach. Estimated treatment and covariate effects with 95% CIs were obtained from the regression models. No formal adjustment for multiple comparisons was planned. Interpretation emphasised the magnitude and precision of the estimated effects, based on point estimates and 95% CIs, rather than statistical significance alone. A two‐sided *P* < 0.05 was considered statistically significant for each outcome. All statistical analyses were performed using R 4.5 [13].

### Primary Outcomes

The following four primary outcomes were studied in this clinical trial: total operative time, overall performance, effort, and frustration ratings.

### Secondary Outcomes

Our secondary outcomes were to evaluate retropulsion during holmium laser stone fragmentation and postoperative stone outcomes between standard and Moses 2.0 laser modalities.

## Results

A total of 171 patients were enrolled with 79 patients randomised to standard holmium laser lithotripsy and 74 to Moses 2.0 laser lithotripsy. In all, 18 patients were excluded from the analysis due to factors such as missing data collection, improper adherence to protocol, or stone exclusion criteria (Fig. 1).

**Fig. 1:**
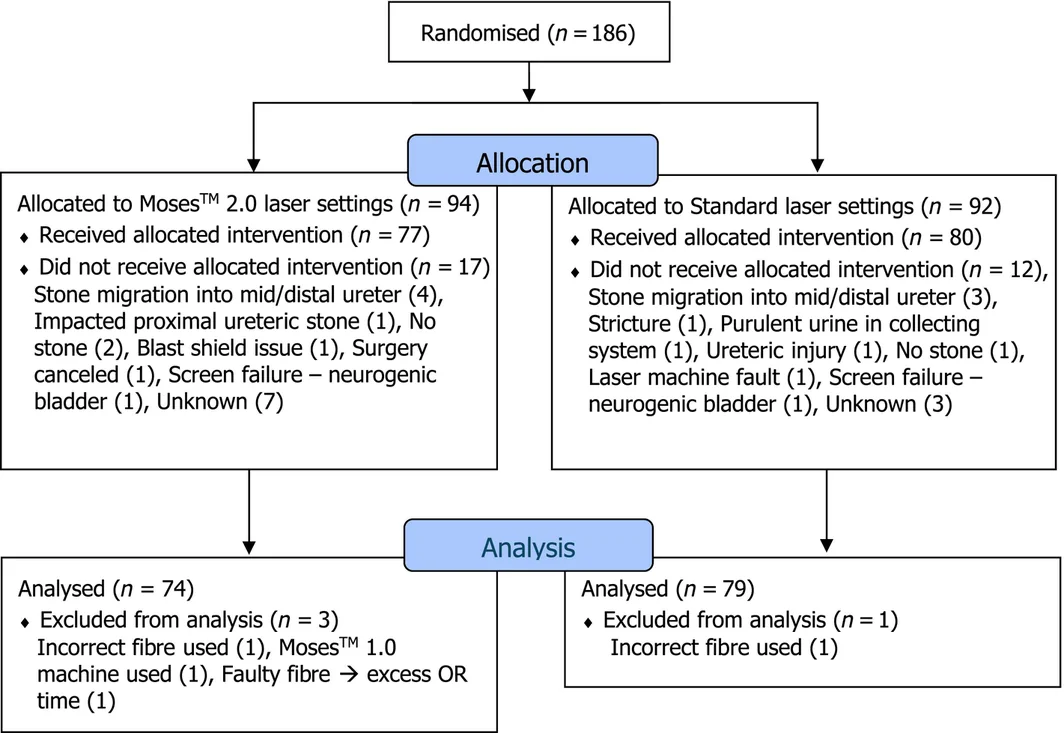
The Consolidated Statement of Reporting Trials (CONSORT) diagram. OR, operating room.

Baseline characteristics were summarised descriptively without formal statistical testing (Table 1). There were no observed differences between clinical, stone, and surgical characteristics between the Standard and Moses 2.0 groups. Stones treated with the Moses 2.0 mode tended to be slightly larger in the coronal dimension (Standard 9.4 mm vs Moses 2.0 10.5 mm) and more often treated with digital ureteroscope (Standard 60% vs Moses 2.0 77%).

**Table 1:** Clinical, stone and surgical characteristics by treatment type.

Characteristic	Standard (*N* = 79)	Moses 2.0 (*N* = 74)
Age, years, median (IQR)	59 (48–72)	61 (50–69)
Race, *n* (%)		
Asian	1 (1)	2 (3)
Black or African American	7 (9)	6 (8)
Caucasian	69 (87)	62 (84)
Latino	1 (1)	3 (4)
More than one race	1 (1)	0 (0)
Native American/Alaska Native	0 (0)	1 (1)
Female, *n* (%)	44 (56)	38 (51)
BMI, kg/m^2^, median (IQR)	31.1 (26.5–34.4)	30.2 (25.7–35.7)
Diabetes mellitus, *n* (%)	18 (23)	16 (22)
Hypertension, *n* (%)	36 (46)	39 (53)
Primary hyperparathyroidism, *n* (%)	1 (1)	2 (3)
Inflammatory bowel disease, *n* (%)	2 (3)	0 (0)
Gastric bypass or bowel resection, *n* (%)	4 (5)	3 (4)
Migraines and/or seizures, *n* (%)	2 (3)	4 (5)
HIV, *n* (%)	0 (0)	2 (3)
Cystinuria, *n* (%)	0 (0)	2 (3)
Cystic fibrosis, *n* (%)	0 (0)	1 (1)
Gout, *n* (%)	5 (6)	3 (4)
ASA class, *n* (%)		
1	3 (4)	1 (1)
2	33 (42)	31 (42)
3	40 (51)	38 (51)
4	3 (4)	4 (5)
Prior stones, *n* (%)	52 (66)	50 (68)
Prior stone surgery, *n* (%)	41 (52)	32 (43)
Laterality, *n* (%)		
Left	44 (56)	39 (53)
Right	35 (44)	35 (47)
Number of stones in renal unit, median (IQR)	1 (1–2)	1 (1–2)
Axial stone size, mm, median (IQR)	9.7 (8.0–11.5)	10.0 (8.0–12.5)
Coronal stone size, mm, median (IQR)	9.4 (8.0–11.0)	11.0 (8.4–13.0)
Hounsfield units, median (IQR)	985 (741–1257)	1125 (825–1338)
Stone location, *n* (%)		
Renal pelvis	22 (28)	24 (32)
Upper pole	12 (15)	11 (15)
Interpolar	9 (11)	9 (12)
Lower pole	41 (52)	31 (42)
Calyceal diverticulum	0	0
Ureteropelvic junction	17 (22)	16 (22)
Hydronephrosis, *n* (%)	27 (34)	29 (39)
Preoperative drainage, *n* (%)		
None	62 (79)	59 (80)
Ureteric stent	17 (22)	14 (19)
Nephrostomy tube	0 (0)	1 (1)
Digital ureteroscope, *n* (%)	47 (60)	57 (77)
Access sheath used, *n* (%)	66 (84)	62 (84)
Stones repositioned, *n* (%)	39 (49)	40 (54)
Location stone treated, *n* (%)		
Upper pole calyx	49 (62)	53 (72)
Interpolar calyx	8 (10)	7 (10)
Lower pole calyx	18 (23)	12 (16)
Renal pelvis	17 (22)	17 (23)
Anti‐platelet continued, *n* (%)	10 (13)	5 (7)
P2Y12 inhibitor continued, *n* (%)	2 (3)	0 (0)
Anti‐coagulant continued, *n* (%)	9 (11)	8 (11)
Active basketing (>2 passes), *n* (%)	3 (4)	2 (3)
Chronic alpha‐blocker, *n* (%)	14 (18)	16 (22)
Alpha‐blocker duration postoperatively, days, median (IQR)	10 (7–14)	9 (7–10)
Stent duration, days, median (IQR)	7 (5–8)	7 (5–10)
Ancillary procedure, *n* (%)	2 (3)	2 (3)
Primary* stone composition, *n* (%)		
COM	43 (54)	33 (45)
COD	4 (5)	6 (8)
CAP	13 (17)	14 (19)
UA	4 (5)	2 (3)
Unknown	15 (19)	19 (26)

ASA, American Society of Anesthesiologists; BMI, body mass index; CAP, calcium phosphate; COD, calcium oxalate dihydrate; COM, calcium oxalate monohydrate; P2Y12 inhibitor, clopidogrel–prasugrel–ticagrelor–etc.; UA, uric acid. Continuous values reported as median (interquartile range [IQR]). Categorical variables presented as number (%).

* Primary means >50% stone composition.

There was no difference in operative times for the Standard and Moses 2.0 mode (*P* = 0.73, Table 2). Significant differences were seen in the laser settings; however, overall settings were similar from baseline laser settings per study protocol. Laser settings were adjusted in about half of all cases regardless of laser mode (*P* = 0.70). There were no significant differences in laser time (*P* = 0.16), energy (*P* = 0.29), or number of times the pedal was pressed (*P* = 0.80) for either laser mode. The laser mode was correctly perceived by the surgeon intraoperatively in 52% of cases for standard holmium laser and 61% for Moses 2.0 (*P* = 0.11).

**Table 2:** Operative times and laser parameters by treatment type.

Variable	Standard (*N* = 79)	Moses 2.0 (*N* = 74)	*P*
Total operation time, min, median (IQR)	36 (30–50)	37 (31–51)	0.73
URS time, min, median (IQR)	28 (21–40)	26 (20–37)	0.47
Lasing time, s, median (IQR)	450 (327–697)	550 (353–780)	0.35
Laser energy used, kJ, median (IQR)	10.2 (6.1–16.0)	11.9 (7.1–17.4)	0.29
Baseline laser settings per protocol	0.2 J–80 Hz short pulse (left pedal) 0.5 J–80 Hz short pulse (right pedal)	0.2 J–80 Hz distance mode (left pedal) 0.3 J–120 Hz contact mode (right pedal)	–
Most common pulse energy, J, median (IQR)	0.3 (0.2–0.4)	0.3 (0.2–0.3)	**0.005**
Pulse energy maximum, J, median (IQR)	0.5 (0.5–0.5)	0.3 (0.3–0.3)	**<0.001**
Most common frequency, Hz, median (IQR)	80 (79–80)	90 (80–100)	**<0.001**
Frequency maximum, Hz, median (IQR)	80 (80–80)	120 (120–120)	**<0.001**
Changed laser settings, *n* (%)	29 (54%)	27 (53%)	0.70
Times laser pedal pressed, *n*, median (IQR)	85 (46–142)	75 (40–137)	0.80
% time lasering, median (IQR)	63 (47–71)	65 (53–78)	0.16
Surgeon blinding, *n* (%)	79 (100)	72 (97)	0.48
Correct setting perceived, *n* (%)	41 (52)	45 (61)	0.11

Continuous values reported as median (interquartile range [IQR]). Categorical variables presented as number (%).

Bold values statistically significant at *P* < 0.05.

### Evaluation of Workload via the NASA‐TLX

When comparing post‐operation surgeon assessments, for all axial stone size categories, surgeons reported that use of the Moses 2.0 laser modality was associated with lower mental, physical, and temporal demands (Fig. 2a). Similarly, surgeons reported that the use of the Moses 2.0 laser modality was associated with higher overall performance, as well as lower effort and frustration levels (Fig. 2b).

**Fig. 2:**
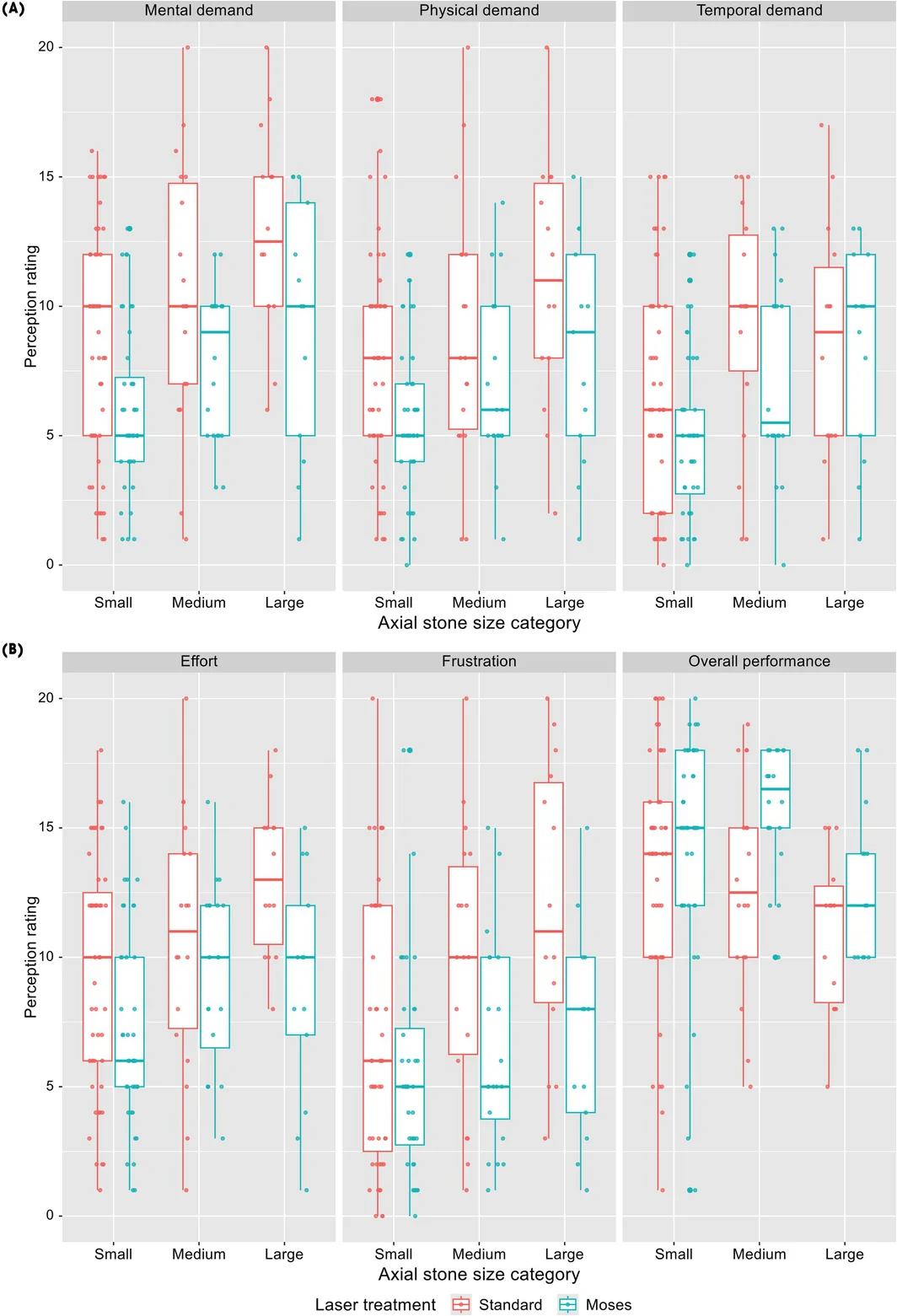
(**A**) Procedure exertion on NASA‐TLX surgeon survey by treatment and axial stone size; (**B**) Satisfaction on NASA‐TLX surgeon survey by treatment and axial stone size. For axial stone size category: Small = 8.0–12.0 mm, Medium = 12.1–16.0, Large = 16.1–20.0 mm.

### Regression Analysis

The interaction between treatment and stone size was not statistically significant and was therefore omitted from the final model for parsimony. In the covariate‐adjusted regression model, total operative time for patients under the Moses laser was on average 0.6 min longer (95% CI 5.5 min shorter, 6.7 min longer) than those under the standard laser, but there was insufficient evidence to suggest that the laser type was associated with total operative time (*P* = 0.85; Table S1). However, use of the Moses 2.0 mode was associated with improved overall performance, lower frustration, and lower perceived effort ratings (Table 3). On average, surgeons using the Moses 2.0 laser achieved performance scores 2.3 points higher (95% CI 0.92–3.7, *P* = 0.001), frustration scores 3.1 points lower (95% CI 1.5–4.7, *P* = <0.001), and effort ratings 3.2 points lower (95% CI 1.8–4.7, *P* < 0.001). Additional covariate results for this analysis can be found in Table S2.

**Table 3:** Regression modelling results for surgeon assessment outcomes.

Predictor/covariate	Outcome of interest					
	Overall performance rating		Frustration rating		Effort rating	
	Treatment effect (95% CI)	*P*	Treatment effect (95% CI)	*P*	Treatment effect (95% CI)	*P*
**Moses 2.0 laser treatment**	**2.3 (0.9 to 3.7)**	**0.001**	**−3.1 (−4.7 to 1.5)**	**<0.001**	**−3.2 (−4.7 to −1.8)**	**<0.001**
Patient age	−0.014 (−0.063 to 0.035)	0.57	0.037 (−0.020 to 0.093)	0.20	−0.006 (−0.056 to 0.045)	0.82
Patient BMI	−0.017 (−0.11 to 0.073)	0.71	0.019 (−0.085 to 0.12)	0.72	0.015 (−0.078 to 0.11)	0.74
Hounsfield units	−0.002 (−0.004 to 0)	0.12	0.004 (0.002 to 0.007)	0.001	0.003 (0.001 to 0.005)	0.004
Number of stones	−0.31 (−0.85 to 0.24)	0.27	0.17 (−0.46 to 0.80)	0.59	−0.28 (−0.84 to 0.29)	0.33
Axial stone size 12.1–16.0 mm*	0.36 (−1.3 to 2.0)	0.67	2.4 (−0.45 to 4.3)	0.016	2.1 (0.41 to 3.9)	0.016
Axial stone size 16.1–20.0 mm	−1.2 (−3.2 to 0.91)	0.27	2.5 (0.082 to 4.9)	0.043	2.4 (0.28 to 4.6)	0.027
Total operative time	0.023 (−0.019 to 0.065)	0.279	−0.013 (−0.062 to 0.035)	0.58	0.028 (−0.016 to 0.071)	0.21
Stones repositioned	0.70 (−0.81 to 2.2)	0.36	−0.39 (−2.1 to 1.4)	0.66	0.47 (−1.1 to 2.0)	0.56
Digital scope	1.0 (−0.60 to 2.6)	0.22	1.1 (−0.74 to 3.0)	0.24	0.43 (−1.2 to 2.1)	0.61
Ureteric access sheath use	−1.4 (−3.2 to 0.49)	0.15	−0.35 (2.5 to 1.8)	0.75	−1.7 (−3.6 to 0.21)	0.080
Lower pole location dusting	−0.13 (−2.1 to 1.9)	0.90	0.98 (−1.3 to 3.3)	0.40	0.38 (−1.7 to 2.4)	0.72

* Reference: 8.0–12.0 mm.

BMI, body mass index. Bold values statistically significant at P < 0.05.

### Postoperative Course

There were no significant differences in rates of postoperative complications between the treatment groups (*P* = 0.54). Clavien–Dindo complications occurred in 18 patients (Standard: 10 patients [13%]; Moses 2.0: eight [11%]) with the majority (Standard: eight patients [10%]; Moses 2.0: five [7%]) being low grade (Grade I or II) (Table S3). There were three patients who had a Clavien–Dindo Grade IIIb complication where a repeat operative procedure was required. In the standard holmium laser group, one patient had pyelonephritis with an obstructing stone fragment requiring stent replacement and one patient had intolerable postoperative stent symptoms requiring repeat URS, where no residual fragments were seen. One patient treated with the Moses 2.0 laser required repeat URS due to symptomatic obstructing stone fragments. In addition, one patient in each treatment group had intensive care unit admission for urosepsis with positive blood cultures (Grade IVa). An additional patient in the Moses 2.0 treatment group had severe postoperative urosepsis with high pressor requirements complicated by limb ischaemia and non‐ST elevated myocardial infarction (Grade IVb).

On postoperative CT, no significant differences in SFRs were observed with no residual fragments in 51% for standard holmium laser and 55% for Moses 2.0 groups (Table S2). There was also no significant difference in stone‐free status based on preoperative stone size (*P* = 0.92).

### Intraoperative Video Review

Blind independent reviewers were only able to correctly determine if standard or Moses 2.0 mode was used about half of the time, indicating that this was no different from a guess (Reviewer A: 51%, Reviewer B: 59%). Variable degrees of retropulsion were noted but were not significantly different for either laser mode (*P* = 0.13, Fig. 3). The majority of cases were noted to have stones smaller than the laser fibre at end of dusting with no observed differences treatment groups (Standard: 98% vs Moses 2.0: 96%, *P* = 0.47).

**Fig. 3:**
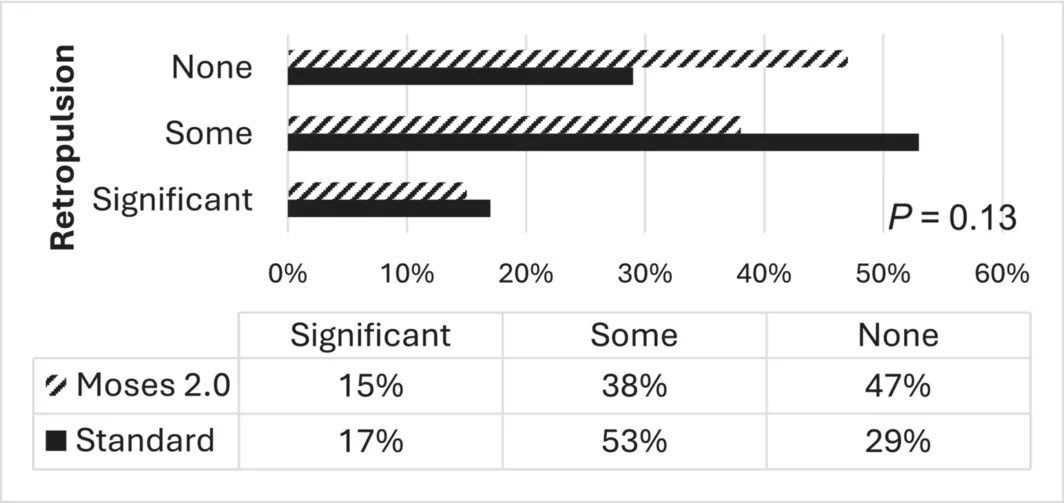
Retropulsion perceived on surgical video review.

## Discussion

Ureteroscopy is the most common treatment modality for renal stones but can be technically demanding. Multiple innovative technologies have been implemented to improve stone treatment efficiency. We designed our study to evaluate the clinical benefit of Moses 2.0 pulse modulation. In our study, Moses 2.0 improved the surgeon's overall performance, effort, and frustration when compared to standard holmium laser settings across all stone sizes in the largest, double‐blind trial to date. However, Moses 2.0 did not clearly reduce operative time, SFRs, or complications.

We evaluated the impact of this new technology on a surgeon's cognitive burden; this is a novel way to evaluate new surgical technology. The impact of laser pulse modulation on surgeon workload has not previously been evaluated using a validated survey. Originally developed for NASA to measure the demands of any task, the NASA‐TLX has been validated in other surgical fields [8, 9]. Stewart et al. [14] reported that robot‐assisted surgery was associated with a 3‐point lower difference in physical demand (median 10 vs 13, *P* = 0.03) when compared to open surgery. In our study, Moses 2.0 resulted in a statistically significant 2‐point improvement in performance as well as 3‐point improvements in frustration and effort.

For all axial stone size categories, surgeons reported that use of Moses 2.0 was associated with lower mental, physical, and temporal demands (Fig. 2a), as well as higher overall performance and lower effort and frustration levels (Fig. 2b) for all stone sizes. This study was not powered for statistical analysis of these subsets of the study population. In our regression model, workload correlates strongly with known predictors of difficult URS such as stones with high Hounsfield units or stones in lower pole that provide a degree of internal validation. When controlling for these same parameters, only Moses 2.0 was significantly associated with improved performance, frustration, and effort (Table 3).

This study did not clearly delineate the cause of the decreased workload reported with use of the Moses 2.0. However, we hypothesise that pulse modulation may offset some of the workload associated with unfavourable stone characteristics. Prior studies with high‐speed photography and *ex vivo* stone models have demonstrated that Moses pulse modulation results in increased efficiency of stone ablation including decreased retropulsion [15, 16] and smaller fragments [17]. Another clinical study reported that subjectively urologists also felt there was less retropulsion with Moses technology [18]. Decreased motion may result in increased energy contact with the stone and/or decreased need to re‐position the ureteroscope. These effects can have a positive impact on effort and frustration, especially with hard‐to‐reach stone locations or a large stone size.

Although traditional objective clinical outcomes have been the focus of clinical trials, the role of surgeon workload is something we believe should be considered when evaluating new technology. Task load of daily clinical duties is now shown to be strongly associated with risk of burnout among a large survey of United States physicians, which included both NASA‐TLX and Maslach Burnout Inventory surveys [19]. Therefore, decreased workload could decrease the risk of burnout. The reduction in workload can be compounded if multiple URSs are performed consecutively. By extension, this could affect patient outcomes; as poorer perceived performance during a harder than anticipated surgery correlates negatively with mental and physical demand across various surgeries as measured by the NASA‐TLX [20]. Highlighting benefits through standardised task load surveys can also be beneficial for capital purchases that may improve surgeon wellness.

Multiple studies have reported variable clinical outcomes with Moses pulse modulation. We found that the results were inconclusive in identifying a difference in operative time with Moses 2.0. Other recent trials comparing Moses to other high‐powered lasers have not shown improvements in traditional surgical benchmarks, similar to our findings. A propensity score matched evaluation of 206 patients treated with a 60 W Moses 1.0 laser vs a conventional 100 W laser using standardised settings (0.4–1  J, 20–40 Hz) did not find a statistically significant improvement in operative time or SFRs with Moses technology [21]. Similarly, a back‐to‐back comparison of consecutive URSs using the Lumenis Pulse 120H with Moses deactivated for the first 66 cases and Moses 1.0 activated for the subsequent 110 cases with the same laser settings in each group (0.3 J, 80 Hz dusting; 0.8 J, 8 Hz fragmentation) showed no significant difference in procedural time, fragmentation/dusting time, lasing time, total energy used, or SFRs [22]. In contrast, another double‐blind trial compared 72 patients with both ureteric and renal stones showed that using Moses 1.0 pulse modulation was associated with decreased fragmentation and procedure time as compared to no pulse modulation; however, stones in this trial were treated with a combination of fragmentation with basket extraction and dusting technique [18].

Differences in results can be secondary to study design. Previous clinical studies have some confounding features including lack of surgeon blinding, highly variable laser power, mix of basketing and dusting techniques, and combined ureteric and renal stone location, could be confounding variables [15, 18, 23, 24, 25, 26]. We designed our study to evaluate the benefits of Moses pulse modulation alone. Our study was limited to renal stones, where stone retropulsion during laser ablation is most evident. We also used a dusting technique on renal stones, which depends on ablation efficiency. Additionally, by comparing Moses technology to short pulse duration in the standard arm, any difference in treatment effect should have been more pronounced. While adjustments in laser settings were allowed and occurred in about half of the cases, the most common setting used per case remained 0.2–0.3 J and 80 Hz regardless of Moses activation. There was also no difference in percentage of time spent lasering observed between the laser modes, further indicating comparable laser treatment conditions.

We acknowledge several limitations of this trial. Notably, the trial did not satisfy the pre‐defined power analysis. The trial was terminated early secondary to widespread use of suction technology revolutionising the URS treatment paradigm. Pure dusting became outside the scope of the standard of care for the tertiary care centres participating in this study. Although we did not reach our intended enrolment, our trial is larger than other previously reported trials. Our sample size in this study with surgical videos for review of retropulsion was limited and an effect may have been detected with a larger study population. It is possible that the modest‐sized stones in our trial were not large enough to result in a significant difference between Moses and standard laser lithotripsy. Every effort was made to blind the surgeon to laser settings in this real‐world clinical trial especially given the subjective nature of the workload secondary outcomes; however, it is possible that experienced surgeons could infer the laser mode. The surgeon's ability to select the laser modality at the time of completing the workload questionnaire was not statistically significant. In this study, the difference in retropulsion was not identified on video review by two blinded independent fellowship‐trained endourologists; however, the scale of the retropulsion and stone fragmentation can be smaller than can be reliably captured on video or perceived by the human eye on review, especially at the high‐power and high‐frequency settings selected in this study. A strength of this trial is that all URSs were performed by attending endourologists to minimise surgical skill as a confounder. However, this may limit generalisability as it is possible that these expert endourologists were already very efficient at laser lithotripsy with a standard laser. It is possible that different outcomes might be observed with Moses 2.0 technology in other practice settings or surgeons with lower treatment volumes.

## Conclusion

In this double‐blind, randomised controlled trial, Moses 2.0 technology did not demonstrate a clear reduction in operative time or improved SFRs compared to standard laser lithotripsy. Although the trial was terminated before the planned enrolment was achieved because of rapid adoption of suction‐assisted URS rendered pure dusting no longer representative of standard practice at the participating tertiary referral centres, this study remains the largest randomised evaluation of pure dusting to date. Notably, Moses 2.0 was associated with decreased surgeon workload across all domains of the NASA‐TLX suggesting potential ergonomic and surgeon wellness benefits. Future studies should evaluate whether these reductions in cognitive and physical workload translate into meaningful downstream effects, particularly when accumulated over multiple procedures and across surgeons with varying levels of expertise.

## Author Contributions

Conceptualisation, design: Naren Nimmagadda, Ryan S. Hsi, Ben H. Chew, Seth K. Bechis, Mitchell Humphreys, Michael E. Lipkin, Manoj Monga, Ojas Shah, Karen L. Stern, Roger L. Sur, Sri Sivalingam, Nicole L. Miller (all equal). Data acquisition: Kimberly A. Maciolek, Naren Nimmagadda, Amy M. Reed, Ryan S. Hsi, Connor M. Forbes, Cyrus Chehroudi, Ezra J. Margolin, Ben H. Chew, Seth K. Bechis, Mitchell Humphreys, Michael E. Lipkin, Manoj Monga, Ojas Shah, Karen L. Stern, Roger L. Sur, Sri Sivalingam, Nicole L. Miller (all equal). Analysis: Naren Nimmagadda, Kimberly A. Maciolek, Connor M. Forbes, Matthew S. Lee, Aaron Lee, Tatsuki Koyama (all equal). Writing: Naren Nimmagadda, Kimberly A. Maciolek, Nicole L. Miller, Aaron Lee, Tatsuki Koyama. Review and editing: all authors contributed equally. All authors approve the submitted version.

## Funding

Foundation grant from the Endourology Disease Group for Excellence (EDGE); unrestricted research grant from Lumenis.

## Disclosure of Interests

Kimberly A. Maciolek, Naren Nimmagadda, Aaron X. Lee, Amy M. Reed, Cyrus Chehroudi, and Ezra J. Margolin report no relevant conflicts of interest. Ryan S. Hsi has NIH grant support. Tatsuki Koyama serves on data safety monitoring board for Vanderbilt University. Connor M. Forbes is a consultant for Boston Scientific, Becton, Dickinson and Company, and Olympus; he has research support from Boston Scientific, Karl Storz and SonoMotion. Matthew S. Lee is a consultant for Boston Scientific and Richard Wolf; he is on the Data Safety Monitoring Board for Butterfly. NN is a joint inventor of a suturing patent and has grand funding from Advanced Research Projects Agency for Health. Ben H. Chew is a consultant for Boston Scientific, ADVA‐Tec, Karl Storz, Olympus Medical, SonoMotion, UroViu, Auris Surgical (Johnson & Johnson), and Laborie. Seth K. Bechis is a consultant for Boston Scientific, Ambu, Dornier, Becton, Dickinson, and Company, Calyxo as well as a speaker for Storz. Mitchell R. Humphreys is a consultant for Boston Scientific, has research grants with ADVA‐Tec, Avvio Medical and SonoMotion and is a data safety monitoring board member for SonoMotion and advisory board member for Boston Scientific, Virtuoso, Becton, Dickinson and Company and Johnson and Johnson and Global, Surgical, Destination, Healthcare Inc. Michael E. Lipkin is a consultant for Boston Scientific. Manoj Monga has financial relationships with Boston Scientific. Ojas Shah has financial relationships with Boston Scientific, Coloplast, Johnson and Johnson, Karl Storz. Karen L. Stern is a consultant for Olympus, Becton, Dickinson and Company, Dornier, Boston Scientific, Applaud Medical and a member of the advisor board for NovoNordisk; she also has investments in SonoMotion. Roger L. Sur is an advisor/consultant for Calyxo, RetroPerc and Boston Scientific as well speaker for Boston Scientific. Sri Sivalingam is a consultant for Karl Storz Endoscopy‐America, Inc., Becton, Dickinson and Company, Cook Medical, and ForTec Medical, Inc. Nicole L. Miller is a consultant for Boston Scientific Corporation, Lumibird, Ventaris Surgical, and Novo Nordisk Inc.

## Ethics and Registration

The study was approved by the institutional review boards at participating centers and was prospectively registered at ClinicalTrials.gov (ClinicalTrials.gov identifier: NCT04505956). All participants provided informed consent.

## Supporting information


**Fig. S1.** The NASA‐TLX questionnaire.
**Table S1.** Regression modelling results for total operation time outcome.
**Table S2.** The SFRs by treatment type.
**Table S3.** Clavien–Dindo complications between treatment groups.

## Data Availability

The data that support the findings of this study are available from the corresponding author upon reasonable request.
