# Supplementary material for: Prospective randomised double‐blind trial: holmium laser lithotripsy with vs without Moses™ 2.0
Source: BJU Int. 2026 Sep 13;138(6):1104–14. doi: 10.1111/bju.70446 (PMC13741120; doi:10.1111/bju.70446)
Supplement: Supplementary file 1 [file BJU-138-1104-s001.docx]

*Supplemental Figure 1: NASA-TLX questionnaire*

**Mental Demand:** How mentally demanding was the task?

1 Very Low

Very High 20

10

**Physical Demand:** How physically demanding was the task?

1 Very Low

Very High 20

10

**Temporal Demand:** How hurried or rushed was the pace of the task?

1 Very Low

Very High 20

10

**Performance:** How successful were you in accomplishing what you were asked to do?

1 Perfect

Failure 20

10

**Effort:** How hard did you have to work to accomplish your level of performance?

1 Very Low

Very High 20

10

**Frustration:** How insecure, discourage, irritated, stressed and annoyed were you?

1 Very Low

Very High 20

10

*Supplemental Table 1: Regression modeling results for total operation time outcome*

| Predictor/Covariate | Estimate | 95% CI | *p* |
| --- | --- | --- | --- |
| **Moses^TM^ 2.0 laser treatment** | **0.61** | **[-5.5, 6.7]** | **0.85** |
| Patient age | 0.18 | [-0.03, 0.40] | 0.092 |
| Patient BMI | 0.13 | [-0.27, 0.52] | 0.53 |
| Hounsfield units | 0.01 | [-0.001, 0.02] | 0.097 |
| Number of stones | 1.7 | [-0.67, 4.1] | 0.15 |
| Axial stone size 12.1-16.0 mm* | -4.8 | [-12.0, 2.6] | 0.20 |
| Axial stone size 16.1-20.0 mm* | 0.82 | [-8.3, 9.9] | 0.86 |
| Stones repositioned | -5.9 | [-13.0, 0.67] | 0.078 |
| Use of a digital ureteroscope | -1.4 | [-8.4, 5.7] | 0.71 |
| Use of a ureteral access sheath | 2.6 | [-5.6, 11.0] | 0.53 |
| Lower pole dusting | 3.2 | [-5.8, 12.0] | 0.47 |

*Reference = 8.0-12.0 mm

*Supplemental Table 2: Stone-free rates by treatment type*

|  | **Standard (N=79)** | **Moses^TM^ 2.0 (N=74)** | ***p-value*** |
| --- | --- | --- | --- |
| Missing | 14 (18%) | 8 (11%) |  |
| Days between surgery and CT | 50 [41, 57] | 47 [40, 59] | 0.48 |
| Residual fragment |  |  | 0.97 |
| Grade A: No residual fragment/stone free | 33 (51%) | 36 (55%) |  |
| Grade B: ≤2 mm | 14 (22%) | 13 (20%) |  |
| Grade C: ≤4 mm | 11 (17%) | 9 (14%) |  |
| Grade D: >4 mm | 6 (9%) | 6 (9%) |  |
| Number of fragments |  |  | 0.85 |
| 1 | 10 (31%) | 15 (50%) |  |
| 2 | 11 (34%) | 8 (25%) |  |
| 3 | 3 (9%) | 2 (7%) |  |
| 4-9 | 4 (13%) | 3 (10%) |  |
| >10 | 4 (13%) | 2 (7%) |  |
| Largest fragment size (mm) | 2.7 [1.8, 3.6] | 2.6 [1.9, 3.6] | 0.77 |

*Supplemental Table 3: Clavien Dindo complications between treatment groups*

|  | **Standard (N=79)** | **Moses (N=74)** | ***p*** |
| --- | --- | --- | --- |
| None | 69 (87.3%) | 66 (89.2%) | 0.54 |
| I | 1 (1.3%) | 3 (4.1%) |  |
|  | - 1 stent symptoms | - 3 renal colic |  |
| II | 6 (8.8%) | 2 (2.7%) |  |
|  | - 2 AUR with foley placement - 3 Pyelonephritis - 1 Urosepsis, +blood cultures, no ICU admission | - 1 AUR with foley placement, UTI - 1 pyelonephritis - 1 urosepsis, +blood cultures |  |
| IIIa | 0 (0%) | 0 (0%) |  |
| IIIb | 2 (2.5%) | 1 (1.4%) |  |
|  | - 1 pyelonephritis with obstructing stone fragment, stent placed - 1 intolerable stent symptoms, repeat URS | - 1 Symptomatic obstructing stone fragments, repeat URS |  |
| IVa | 1 (1.3%) | 1 (1.4%) |  |
|  | - 1 ICU admission for urosepsis, +blood cultures | - 1 ICU admission for urosepsis, +blood cultures |  |
| IVb | 0 (0%) | 1 (1.4%) |  |
|  |  | - 1 urosepsis requiring pressors, stent replaced, NSTEMI |  |

AUR = acute urinary retention; + = positive; URS = ureteroscopy; NSTEMI = non-ST elevation myocardial infarction
